# A Metabonomics Investigation into the Therapeutic Effects of BuChang NaoXinTong Capsules on Reversing the Amino Acid-Protein Interaction Network of Cerebral Ischemia

**DOI:** 10.1155/2019/7258624

**Published:** 2019-03-20

**Authors:** Jing Xu, Xin Liu, Liyu Luo, Liying Tang, Na Guo, Mengting Liu, Hongmei Li, Fangbo Zhang, Yi Zhang, Defeng Li, Ye Zhao, Hongwei Wu, Hongjun Yang

**Affiliations:** ^1^Institute of Chinese Materia Medica, China Academy of Chinese Medical Sciences, Beijing 100700, China; ^2^School of Chinese Materia Medica, Beijing University of Chinese Medicine, Beijing 100029, China; ^3^School of Pharmaceutical Science and Technology, Tianjin University, Tianjin 300072, China; ^4^Experimental Research Centre, China Academy of Chinese Medical Sciences, Beijing 100700, China

## Abstract

**Background:**

Amino acids (AAs) in cerebrospinal fluid (CSF) play a pivotal role in cerebral ischemia (CI). BuChang NaoXinTong Capsules (BNC) are widely prescribed in Chinese medicine for the treatment of cerebrovascular and cardiovascular diseases.

**Methods:**

In order to investigate the therapeutic effects and pharmacological mechanisms of BNC on reversing CI from a system level, an amino acid-protein interaction imbalanced network of CI containing metabolites of AAs, key regulatory enzymes, and proteins was constructed for the first time. Furthermore, a novel method for detecting the ten AAs in CSF was developed by UPLC-QQQ-MS in an effort to validate the imbalanced networks and the therapeutic effects of BNC via analysis of metabolites.

**Results:**

Based on a middle cerebral artery occlusion (MCAO) rat model, the dynamic levels of amino acids in CSF 3, 6, 12, and 24 h after MCAO were analyzed. Up to 24 h, the accumulated nine AA biomarkers were found to significantly change in the MCAO group compared to the sham group and exhibited an obvious tendency for returning to baseline values after BNC treatment. In addition, based on the imbalanced network of CI, four key enzymes that regulate the generation of BNC-mediated AA biomarkers were selected and validated using an enzyme-linked immunosorbent assay and western blotting. Finally, aromatic-L-amino-acid decarboxylase (AADC) was found to be one of the putative targets for BNC-mediated protection against CI.

**Conclusion:**

This study provides new strategies to explore the mechanism of cerebral ischemia and help discover the potential mechanism of BNC.

## 1. Introduction

Stroke is the third leading cause of death after cardiac ischemia and cancer, often resulting in devastating and crippling health conditions. Here, ischemic stroke accounts for about 90% of all stroke cases [1–3]. Cerebral ischemia (CI) is caused by thrombotic or embolic occlusion of a cerebral artery and often leads to neurological malfunctions that may result in irreversible neurological damage or death [4]. Frequently, a significant treatment interruption exists during which no means of effective medical management is available for patients with CI. Moreover, despite numerous clinical trials conducted to salvage cells from death, no significant breakthrough has been made to improve the overall outcome of stroke patients [5].

BuChang NaoXinTong capsules (BNC), as a well-known traditional Chinese medicine (TCM) composed of powder from 16 medicinal herbs or animal powders [6, 7], are approved by the China Food and Drug Administration (CFDA) and recorded in the *Chinese Pharmacopoeia* [8]. As a standard product, BNC has widely been used for the treatment of cardiovascular and cerebrovascular diseases in clinics [9–11]. Chemically, a total of 178 components mainly including flavonoids, triterpenoid saponins, and phenolic acids, have been identified using HPLC-MS in our previous reports [6]. Although previous studies have shown that BNC have various beneficial activities such as antiatherosclerotic through the reduction of lipids, preventing platelet aggregation, antioxidant activity, inhibiting inflammation, and protecting neurons [12–17], the complex mechanisms of BNC are not yet well understood and need to be studied from the system level. Investigations of the complex pharmacological mechanism of BNC may help develop effective treatments and novel therapeutic targets against cerebral ischemia. Recently, the network pharmacology for TCM has become increasingly popular. Network pharmacology analysis technologies based on outcomes of omics data or relative bioinformatics represent a powerful and appropriate method providing interpretations of the complex mechanism of TCM from the system level [18–20]. However, the biological network validation, particularly dynamic validation, represents a key process for interpreting the pathological and TCM-mediated mechanism. Compared with other omics data, metabolites as the bottom signals in the biological information flow exhibit a magnifying feature and may reflect situations in biosystems in real time. Therefore, the dynamic validation of a biological network via analysis of metabolites may help in the discovery of real biomarkers or relative potential targets.

Amino acids, being the basic units of the proteins, comprise the second-largest component of human muscles, cells, and other tissues. Some AAs perform critical roles in cell signaling, biosynthesis, transportation, and key metabolic pathways. Altered levels of amino acids in biological fluids have been found to be closely related to many diseases, e.g., neurological diseases, liver diseases, and stroke [21]. Most neurotransmitters are amino acids; moreover, energy deprivation induced neurotransmitter release which indicated that altered levels of AAs do play a vital role after CI [22]. CSF is a clear, colorless body fluid that can be found in the central nervous system and is generally produced by the choroid plexus. CSF is mostly segregated from the peripheral circulation by the blood-brain barrier and is critical to reflect the metabolic status as well as the biochemistry of brain disorders *in vivo* [23–27]. A growing pool of evidence has already shown that ischemic injury characterized by low oxygen and insufficient glucose supply induces changes in AA concentrations in CSF such as alanine, valine, and phenylalanine [22, 28–31]. However, in these studies, only several amino acids in CSF have been analyzed and dynamic analysis is usually not provided, ultimately limiting the study of amino acids in pharmacological mechanisms and clinical research. Quantitative analysis of amino acid in biological samples is a traditional technology. As most amino acids do not have sufficient UV signals for their detection, the samples are usually derivatized with derivatization reagents before analysis. Considering time-consuming and low sample throughput of derivatization, in recent years, HPLC-MS is the most used detection technique for amino acid analyses because of its high sensitivity and high selectivity and because no analyte baseline separation is required. When utilizing MS for the detection of amino acids, derivatization is not necessary, and the sample preparation can be simplified. As for CSF samples, the analysis method for multi-amino acids was mainly using derivatization [32–34]. Although there are some reports about the detection of AAs by HPLC-MS/MS, the number of AAs detected in the CSF was fewer and limited which cannot fully reflect the levels of AAs in CSF [21, 35–37].

In this study, we developed a comprehensive approach for understanding the pharmacological mechanisms of BNC acting on CI. Firstly, in order to interpret the complex mechanism of TCM against CI, an AA-protein interaction imbalanced network of CI was constructed using related biological information databases and research studies in the literature. Furthermore, a rapid, sensitive, accurate, and specified method for the quantification of amino acids without derivatization in CSF was developed by UPLC-QQQ-MS in an effort to validate imbalanced networks and therapeutic effects of BNC via analysis of the metabolite levels. In this method, ten AAs including alanine (Ala), valine (Val), taurine (Tau), leucine (Leu), isoleucine (Ile), glutamine (Gln), glutamic acid (Glu), phenylalanine (Phe), (S)-tyrosine (Tyr), and tryptophan (Trp) in CSF were accurately detected. Based on a middle cerebral artery occlusion (MCAO) rat model, the dynamic levels of the amino acids in CSF were analyzed. Meanwhile, the regulating effect of BNC on the amino acids was also studied. Subsequently, based on the imbalanced AA-protein interaction network of CI, relative enzymes including aromatic-L-amino-acid decarboxylase (AADC), glutamate oxaloacetate transaminase 1 (GOT1), glutamate oxaloacetate transaminase 2 (GOT2), and tyrosine aminotransferase (TAT) were found to regulate the generation of BNC-mediated AA-biomarkers and tested using enzyme-linked immunosorbent assays (ELISA) and western blotting. The latter helped further identify potential targets and preventive effects of BNC on cerebral ischemia.

## 2. Materials and Methods

### 2.1. Animals, Chemicals, and Reagents

Adult male Sprague-Dawley rats weighing 230 ± 10 g were obtained from the Animal Breeding Centre of Beijing Vital River Laboratories Company (Beijing, China). The project identification code was 20162005. All animals were housed at 22 ± 2°C with a relative humidity of 50 ± 10% and a 12 h light/12 h dark cycle. The animals had free access to water and fodder (Beijing Keaoxieli Co. Ltd.). All experimental animal procedures were approved by the China Academy of Chinese Medical Sciences' Administrative Panel on Laboratory Animal Care and performed in accordance with institutional guidelines and ethics of the committee as part of the China Academy of Chinese Medical Sciences (February 1^st^, 2016).

Ten AA standards including alanine, valine, taurine, leucine, isoleucine, glutamine, glutamate (glutamic acid), phenylalanine, (S)-tyrosine, and tryptophan (purity > 99%, all) were purchased from Sigma-Aldrich (St. Louis, MO, USA). Acetonitrile and methanol were provided from Fisher Scientific (Shanghai, China). Formic acid, ammonium formate, and phenylalanine-d5 (internal standard (IS)) were purchased from Toronto Research Chemicals (YTO, Canada). All chemicals used throughout this study were of HPLC grade unless stated otherwise. BNC (batch number: 140156) was provided from Buchang Pharma Co. Ltd.

### 2.2. Construction of CI Imbalanced Network of AAs-Enzymes-CI-Related Proteins

In order to investigate the relationship between metabolites and proteins after cerebral ischemia, a CI imbalanced network of AAs-enzymes-proteins was constructed. Our protocols included the following four main steps: (1) AAs associated with cerebral ischemia were collected from articles published from 1992 to 2016 [38]. (2) The AAs were entered into the Human Metabolome Database (HMDB), and the corresponding HMDB IDs were retrieved. Then, the regulatory enzymes related to each AA were collected and the common key enzymes related to two or more AAs were selected. (3) The proteins, associated with ischemia as potential targets, were collected by Human Phenotype Ontology (HPO) and Online Mendelian Inheritance in Man (OMIM) database. The ENTREZ GENE ID of the proteins was converted to OFFICIAL GENE SYMBOL using DAVID Bioinformatics Resources [39]. Then, the proteins from the two databases were combined and reweighed. (4) The regulatory enzymes and proteins associated with cerebral ischemia were entered into the Search Tool (STRING database) for the retrieval of protein-protein interactions (PPI) [40]. Only when the PPI score between the protein and enzyme was greater than 0.7, the interaction was confirmed, further providing correlations between the enzymes and cerebral ischemia. Finally, Cytoscape (version 13.6) was applied to visualize the networks containing relationships between AAs, enzymes, and proteins.

### 2.3. MCAO Surgery and Drug Administration

In this study, a permanent MCAO model was applied. According to the reported methods described previously, MCAO surgery was carried out by intraluminal occlusion using a monofilament [41, 42]. 72 rats were randomly divided into three groups: sham operation (sham), MCAO model group with water treatment (MCAO), and MCAO group with BNC treatment (BNC, 220 mg kg^−1^ d^−1^ dosages). The MCAO group was randomly divided into the following subgroups: MCAO_3h_ (3 hours after MCAO), MCAO_6h_ (6 hours after MCAO), MCAO_12h_ (12 hours after MCAO), and MCAO_24h_ (24 hours after MCAO). Six rats were in each subgroup. The sham and BNC groups were also divided into four subgroups according to the surgery time which was the same with the MCAO subgroup. The drugs were orally administered twice a day at 8 am and 8 pm for 5 days. On the sixth day, one hour after the last oral administration in the morning, cerebral ischemia was induced by MCAO. Sham rats were subjected to the same procedures except for nylon filament insertion into the common carotid artery.

### 2.4. Evaluation of MCAO Model and BNC Effect

To evaluate the protective effect of BNC against cerebral ischemia, the neurological function and infarct area were measured. After MCAO operation, the neurological function was evaluated blindly by Longa's Neurological Severity Score [41]. Then, all rats were anesthetized with 10% chloral hydrate at 3, 6, 12, and 24 h after MCAO, respectively. Five coronal sections of the brain (1 mm thickness) were cut, and the slices were stained with 0.5% 2,3,5-triphenyltetrazolium chloride (Sigma, St. Louis, MO, USA) for 15 min at 37°C. After staining with TTC, the normal tissue was stained in a rose red color while the infarct tissue was white. Finally, numeric images were captured for quantification of the infarct volume. The infarct volume of each slice was calculated as infarct area × thickness (1 mm). The summation of the infarct volumes for all brain slices was defined as the total infarct volume.

### 2.5. CSF Sample Collection

After anesthetizing the rats 3, 6, 12, and 24 h after MCAO operation, before evaluation of infarct volume, a depressible surface with the appearance of a rhomb between the occipital protuberances and the spine of the atlas was exposed. The blunt end of the needle was inserted into the cisterna magna, and the other end of the PE-50 tubing was connected to a collection syringe. The CSF samples were collected and stored at -80°C before analysis.

### 2.6. Analytical Method Development of Detecting AAs in CSF by LC-MS/MS

#### 2.6.1. CSF Sample Preparation

After freeze-thawing cycles in an ice bath, an aliquot of 10 *μ*L CSF was mixed with 985 *μ*L of the initial mobile phase and 5 *μ*L of IS (phenylalanine-d5, 200 ng/mL). The mixture was then vortexed for 30 s prior to analysis and deproteinized by centrifugation at 4°C (21,130 g for 10 min), and 5 *μ*L of the supernatant was subjected to UPLC-MS analysis for AA analysis.

#### 2.6.2. Chromatographic and Mass Spectrometric Conditions

Analysis was performed on a Waters Acquity UPLC (Waters Corporation, Milford, MA, USA) instrument consisting of a dual pump, an online degasser, an autosampler, and a thermostatically controlled column. Chromatographic separation was carried out at 40°C on an ACQUITY UPLC BEH amide column (100 mm × 2.1 mm, 1.7 *μ*m, Waters™, USA) with Phenomenex SecurityGuard™ ULTRA. The mobile phase consisted of solvent A (water containing 2 mM ammonium formate and 0.2% formic acid) and solvent B (acetonitrile containing 0.2% formic acid) with a gradient elution (85% A at 0-0.5 min, 85-80% A at 0.5-1 min, 80-76% A at 1-5 min, 76-50% A at 5-5.5 min, and 50-85% A at 5.5-6 min). The reequilibration time of the gradient was 2 min. The flow rate of the mobile phase was 0.3 mL/min. The autosampler was kept at 4°C, and the injection volume was 5 *μ*L.

A Waters Xevo TQ-S triple Quadrupole equipped with an ESI source was used in the positive ion mode and detected by scheduled multiple reaction monitoring (MRM). Ion transitions and retention times for the detection of amino acids are shown in Table 1. Data was acquired using MassLynx 4.1 software and processed by TargetLynx (Waters Corp., Milford, MA, USA). The obtained data was then exported to Excel (2010 Edition, Microsoft Corporation) for further calculations. The compounds were quantified using an internal standard method. The MS parameters for ten amino acids and IS are shown in Table 1.

#### 2.6.3. Method Validation

*(1) Linearity, Sensitivity, and Carryover*. Individual standards (~5 mg) were prepared by dissolving the solids in 5 mL of distilled water. A concentration series of standards from 0.1 to 1000 ng mL^−1^ was prepared by serial dilution with the initial mobile phase consisting of 15% acetonitrile (containing 0.2% formic acid) and 85% water (containing 2 mmol L^−1^ ammonium formate and 0.2% formic acid). The concentration series of standards was used for the plotting of calibration curves and quality control (QC) standards. Before injection, 995 *μ*L of each standard was mixed with 5 *μ*L of IS (phenylalanine-d5, 200 ng/mL). Therefore, the content of IS in each standard was the same as in the prepared CSF sample. Calibration curves were generated using the peak area ratios of ten AAs to IS on the *y*-axis and the corresponding nominal concentrations of ten AAs on the *x*-axis. The sensitivity was evaluated using limits of detection (LOD) and limits of quantification (LOQ) which were determined with the corresponding standard solution at a signal-to-noise (S/N) ratio of ~3 and ~10, respectively. As the concentration range of quantitation was wide in this study, carryover was investigated. The procedure of carryover was to inject a vehicle blank sample following the injections of the standards with upper limit of quantitation (ULOQ) concentrations.

*(2) Precision and Accuracy*. The intra- and interday precisions were determined by analyzing the standard solution containing the ten analytes at intermediate concentration levels, with six daily repetitions over seven consecutive days. The relative standard deviation (RSD) was used as a measure of precision. The accuracy of the developed method was evaluated by recovery experiments with all ten AAs. The recovery was determined by spiking a selected sample. First, the contents of the ten analytes in the sample were calculated according to their respective calibration curves, before spiking six sample aliquots with about identical amounts of the reference compound mixture. Then, the thus fortified samples were prepared and analyzed as described above. The % recovery of the analyte recovery was calculated as follows: recovery (%) = (amount found − original amount)/amount spiked × 100%, and RSD (%) = (SD/mean) × 100%.

*(3) Stability and Repeatability*. To assess freeze-thaw stability, a series of parallel samples from the same representative sample was subjected to three freeze-thaw cycles consisting of thawing samples at room temperature (15~20°C) for at least one hour, vortexing, and then refreezing for at least 12 hours at -80°C. After three freeze-thaw cycles, the samples were analyzed using freshly prepared calibration standards. Considering that the samples were placed in an ice bath and due to the long preparation time as a consequence of large sample numbers, the short-term stability was evaluated by analyzing the samples that were kept in an ice bath for 0, 1, 3, and 6 h before preparation, respectively. For the repeatability assay, six independent samples prepared from the homogenate sample were extracted and analyzed in parallel for the evaluation of repeatability. The RSD was taken as a measure of stability and repeatability.

### 2.7. ELISA and Western Blotting

In order to further validate the imbalanced network of CI and in an effort to investigate the putative targets for cerebral ischemia and BNC, four enzymes of the BNC-mediated AA markers in CSF were assayed using ELISA kits from MyBioSource Inc. (Wuhan, China). According to the manufacturer's instructions, four ELISA kits including rat aspartate aminotransferase, mitochondrial (GOT2) ELISA kit, rat aromatic-L-amino acid decarboxylase (AADC) ELISA kit, glutamate oxaloacetate transaminase 1 (GOT1) ELISA kit, and tyrosine aminotransferase (TAT) ELISA kit were applied. Each kit consisted of a 96-well plate into which a specific antibody against a target protein was immobilized. The target protein in CSF was recognized by the antibody, followed by incubation with a horseradish peroxidase-conjugated secondary antibody for colorimetric quantification. The reactions were carried out in triplicate for each sample. The protein level of AADC was further analyzed by western blotting. Briefly, the CSF proteins were resolved on 10% SDS-PAGE gels and transferred onto PVDF membranes subsequently. Western blotting was performed as described in the manufacturers' protocol. Equal proteins were incubated in primary antibody (the antibody detail: ab3905) followed by horseradish peroxidase-conjugated anti-rabbit secondary antibodies and then detected by ChemiDoc XRS+ Molecular Imager (XRS: X-ray spectrometer) (Bio-Rad, USA). Finally, the results were analyzed by one-way analysis of variance.

### 2.8. Data Processing and Statistical Analysis

Multivariate analysis was performed using SIMCA-P 12.0 software. Principal component analysis (PCA) was first used as an unsupervised method to visualize the overall differences for sham, model, and BNC at 3, 6, 12, and 24 h after MCAO, respectively. All values are presented as means ± standard error of the mean. Statistical significance was determined by one-way ANOVA followed by Tukey's multiple comparison test or Student's *t*-tests. A value of *p* < 0.05 was considered statistically significant.

## 3. Results and Discussion

### 3.1. AA-Enzyme-Protein Interaction of CI

**Figure 1:**
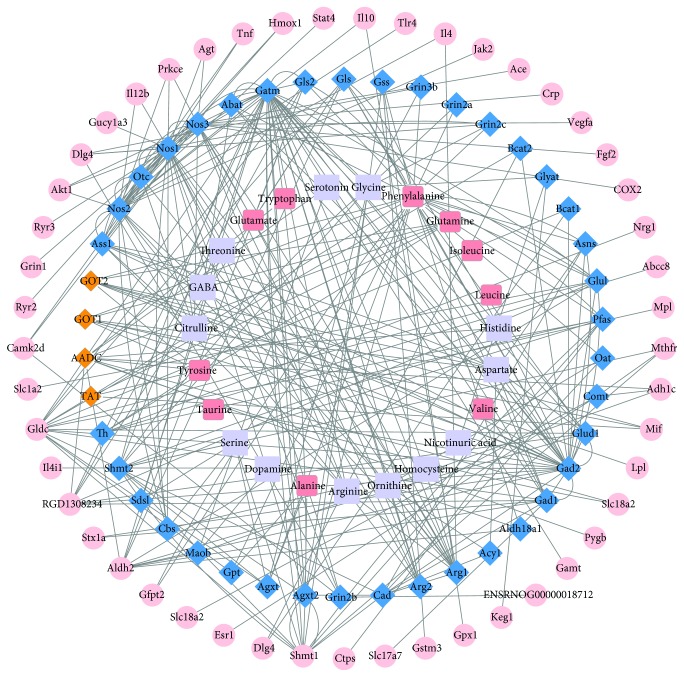
The CI imbalanced network of AAs-enzymes-CI-related proteins. Light purple square: 23 AAs that were collected after CI from articles published from 1992 to 2016; peach square: the ten AAs in CSF detected in this study; blue diamond: key regulatory enzymes that were involved in the regulation of two or more metabolisms of AAs; orange diamond: four key enzymes regulating the generation of BNC-mediated AA-biomarkers measured by ELISAs and western blotting in CSF; pink circle: the proteins associated with CI.

In total, 23 AAs associated with cerebral ischemia were collected as described in our previously reported study by Liu et al. summarizing significant metabolite changes after cerebral ischemia from articles published from 1992 to 2016. The 23 AAs were collected from various tissues including plasma, serum, CSF, cortex, hippocampus, striatum, thalamus, midbrain, white matter, pineal body, and olfactory bulb. Using HMDB, among the 23 AAs (alanine, dopamine, serine, tyrosine, citrulline, GABA, threonine, glutamate, tryptophan, serotonin, glycine, phenylalanine, glutamine, histidine, aspartate, nicotinuric acid, homocysteine, ornithine, arginine, taurine, valine, leucine, and isoleucine), 67 key enzymes which were involved in the regulation of two or more AAs were selected. Using HPO and OMIM databases, 393 proteins associated with cerebral ischemia were collected initially. Based on the above data of 67 key enzymes and 393 proteins, with the use of the STRING database for the retrieval of protein-protein interactions (PPI score > 0.7), 49 proteins and 42 key regulatory enzymes were finally selected. Thereafter, the CI imbalanced network of AAs, enzymes, and proteins was constructed by Cytoscape 13.6 as shown in Figure 1 (the detailed information of the network including 23 AAs, 42 key regulatory enzymes, and 49 proteins is provided in the Supplementary Information section, Table S1). In general, network topological analyses are used to confirm the key targets based on the topological property such as “degree,” “betweenness,” “closeness,” and “*K* value” [43, 44]. However, in this study, a novel strategy of dynamic validation of the imbalance network was used from metabolite levels. Via preliminary experiments, a developing method was attempted to detect all 23 AAs in the network. As the 23 AAs in the network were collected from various tissues such as plasma, serum, CSF, and other neurotissue, the levels of some AAs in CSF were too low to be detected accurately even with derivation or solid-phase extraction (SPE) to prepare the samples. Finally, a rapid, sensitive, accurate, and specified method for detecting ten AAs without derivatization in CSF was developed by UPLC-QQQ-MS.

### 3.2. Evaluation of the Pharmacological Effects of BNC on MCAO Rats

**Figure 2:**
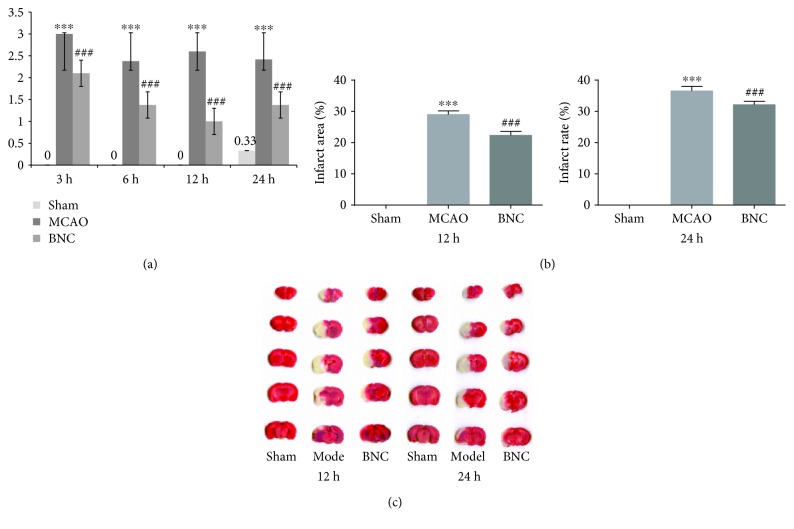
Neurological score by BNC pretreatment: neurobehavioral score (a), infarct area (b), and TTC staining of the brain (c). ^∗^ *p* < 0.05, ^∗∗^ *p* < 0.01, and ^∗∗∗^ *p* < 0.001 in the MCAO group versus the sham group; ^#^ *p* < 0.05, ^##^ *p* < 0.01, and ^###^ *p* < 0.001 in the BNC group versus the MCAO group.

To experimentally investigate the potential biomarkers in the BNC-mediated protection against cerebral ischemia, first, the pharmacological effects of BNC on MCAO mice were evaluated. In our previous study, the effect of the different doses of BNC were investigated [29]; here, we chose a medium dose (220 mg kg^−1^ d^−1^ dosages) to do further research. As shown in Figure 2(a) 3, 6, 12, and 24 h after MCAO operation, Longa's Neurological Severity Score in the MCAO groups revealed remarkable ischemic injuries (*p* < 0.001). However, BNC capsules decrease these scores, indicating improved neurological function in MCAO rats (*p* < 0.001). A similar phenomenon could also be observed in the cerebral infarct area as part of the serial coronal brain sections. The MCAO-induced ischemia produced a marked infarct area in the serial coronal brain sections. TTC staining of the relevant rat tissues after BNC treatment showed a significantly lower degree of ischemic injury compared to the MCAO group, particularly at 12 and 24 h after MCAO. As shown in Figures 2(b) and 2(c), the corresponding infarct volumes demonstrated that BNC exhibited significant protective effects against MCAO-induced ischemic injury. Taken in concert, all of these experimental results confirmed a reliable protective effect of BNC on ischemic stroke.

### 3.3. Method Validation

#### 3.3.1. Linearity, Sensitivity, and Carryover

**Figure 3:**
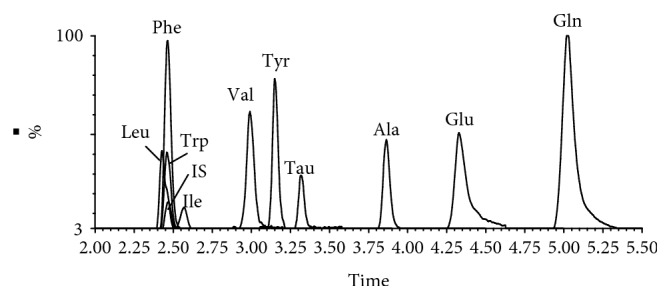
Typical overlap chromatograms of 10 AAs and internal standard. Alanine (Ala), valine (Val), taurine (Tau), leucine (Leu), isoleucine (Ile), glutamine (Gln), glutamate (Glu), phenylalanine (Phe), (S)-tyrosine (Tyr), tryptophan (Trp), and phenylalanine-d5 (internal standard (IS)).

To validate this imbalanced network from the different levels of metabolites, all AAs in the network were attempted to be determined. According to a preexperiment, a novel analytical method of accurately detecting ten AAs including alanine, valine, taurine, leucine, isoleucine, glutamine, glutamate, phenylalanine, tyrosine, and tryptophan in CSF was developed and validated by UPLC-QQQ-MS.  Figure 3 shows the typical overlap in the extracted ion chromatograms (EIC) of the ten detected AAs in CSF. The linear response of the calibration curves was determined by preparation of a set of standards. As shown in Table 2, a correlation coefficient (*r*) for all analytes was obtained from 0.9834-0.9995 which indicated a good fit of the regression model over the respective concentration ranges. The instrument provided consistent results throughout 1000 injections, without the need for extra cleaning or maintenance. Furthermore, the method exhibited an excellent sensitivity based on signal-to-noise (S/N) ratio. For all analytes, the LOD was in the range of 0.7–110 pg/mL (at S/N > 3) and the LOQ was 7-1100 pg/mL (S/N > 10), a finding that proved to be a significant improvement over other existing methods. Specifically, the lower limit of detection of leucine was 0.7 pg/mL and the corresponding lower limit of quantification was 7 pg/mL. As for carryover evaluation, the vehicle blank sample injected following the ULOQ standard injection has no significant peaks at the retention times of all the analytes. Considering that the carryover measurement can be affected by its position in the sampling sequence due to adsorptive carryover issues, vehicle blank samples were injected at regular intervals (every ten samples) throughout the analytical run. In addition, as the ion pairs including parent ion and daughter ion (as shown in Table 1) detected by MRM between IS and the analytes were all different, there is no significant crosstalk observed between IS and analytes in this method.

#### 3.3.2. Precision and Accuracy

The intra- and interday precision and accuracy of the method were evaluated from QC samples and can be found summarized in Table 3. The precision of the present method was in agreement with the criteria for the analysis of biological samples where the RSD was determined to be less than 5%. The average recoveries of the ten AAs in the fortified samples were from 80.3% to 115.3%, and the corresponding RSDs were all determined to be less than 8%, indicating a suitable accuracy for the determination of 10 AAs in the CSF.

#### 3.3.3. Sample Stability and Repeatability

The stability of all analytes was evaluated by analyzing the CSF samples during sample collection and handling. The results of all stability tests are shown in Table 3. Overall, a good stability of all analytes in CSF after three freeze-thaw cycles (RSD < 5%) was demonstrated. In the short-term stability test of six hours in an ice bath, the RSD of the analytes was within 5% except for valine with a RSD value of 5.86, suggesting that the sample exhibited high stability in the ice bath before preparation. To confirm repeatability, six parallelly prepared CSF samples were analyzed and the RSDs of the ten detected AAs were 0.59%–7.99%. Taken in concert, the results outlined above indicate that this analytical method was accurate as well as stable and reproducible, within acceptable limits, and could be used to analyze AAs in CSF.

### 3.4. Dynamic Validation of the Amino Acid Levels in the Imbalanced Networks of CI and Effects of BNC

**Figure 4:**
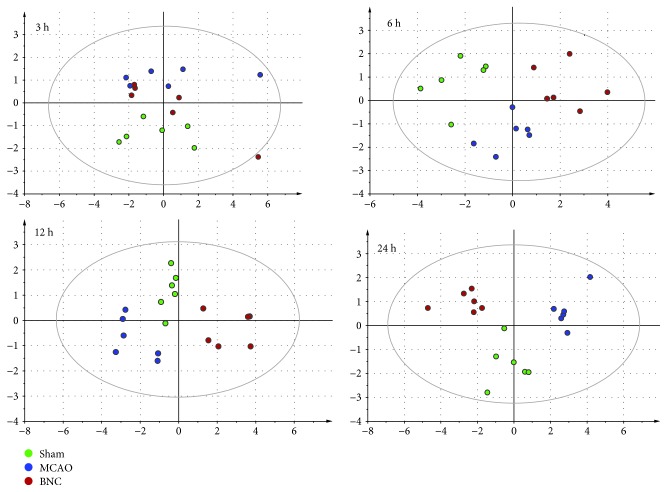
The score plot using the first two principal components for the PCA model of sham, MCAO, and BNC-treated rats. Green circle: sham; blue circle: MCAO; red circle: BNC.

To investigate the global metabolism variations of AAs, PCA was firstly used to evaluate the observations acquired at different time points after MCAO. PCA, an unsupervised pattern recognition method for handling metabonomics data, has been shown to be able to classify the AA metabolic phenotypes based on all imported samples. As shown in the PCA score plot (cf.  Figure 4), an overview of all samples in the data at different time points could be observed. In general, a grouping trend between the sham group, MCAO group, and BNC-treated group could be observed at 3, 6, 12, and 24 h after MCAO, respectively. Furthermore, the longer the ischemia time, the more clear the distinction between the three groups. Furthermore, the corresponding characteristics (R^2^X and *Q* ^2^) of the PCA models, which represent the variance and predictive ability of the model, became greater with ischemia time (3 h: R^2^X, 0.612; *Q* ^2^, 0.143; 6 h: R^2^X, 0.648; *Q* ^2^, 0.279; 12 h: R^2^X, 0.764; *Q* ^2^, 0.287; and 24 h: R^2^X, 0.806; *Q* ^2^, 0.594). This finding suggests that with longer ischemia time, the level changes of AAs in the CSF became more significant. At the same time, this observation indicated that upon administration of BNC, an effect on the MCAO-induced changes in AA levels could be observed even though the trajectory of the BNC groups did not return back to normal levels (i.e., sham groups).

All AA contents in the different groups were shown as mean ± SD (cf.  Table 4). Consistent with the above conclusion, there were 2, 2, 5, and 8 AAs exhibiting significant content changes corresponding to the MCAO_3h_, MCAO_6h_, MCAO_12h_, and MCAO_24h_ group compared with the corresponding sham groups, respectively. Until 6 h after MCAO, there were only three AAs of glutamate, tyrosine, and tryptophan exhibiting a significant incremental change (*p* < 0.01) compared to the sham groups. Up to 24 h after MCAO, except for taurine, there were a total of nine AAs (alanine, valine, leucine, isoleucine, glutamine, glutamate, phenylalanine, tyrosine, and tryptophan) exhibiting significant changes in the MCAO groups compared to the corresponding sham groups at different time points.

**Figure 5:**
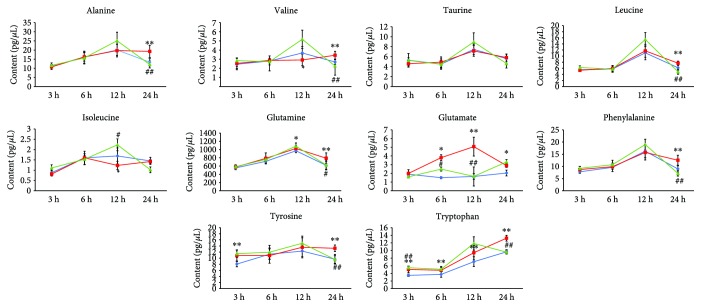
The line chart of 10 amino acid levels in CSF. Blue: sham group; red: MCAO model group; green: BNC-treated group. ^∗^ *p* < 0.05, ^∗∗^ *p* < 0.01, and ^∗∗∗^ *p* < 0.001 in the MCAO group versus the sham group; ^#^ *p* < 0.05, ^##^ *p* < 0.01, and ^###^ *p* < 0.001 in the BNC group versus the MCAO group.

Glutamate as an excitatory amino acid features excessive expression after ischemia and may cause neurotoxic effects on the nervous system [45, 46]. In this study, we found that the level of glutamate in the MCAO group exhibited a significant increase (*p* < 0.01) compared with the sham group as early as 6 h and up to 24 h after cerebral ischemia. The highest level of glutamate in CSF appeared at 12 h after cerebral ischemia. Unlike glutamate, the level of alanine, an inhibitory amino acid, did not exhibit a significant increase until 24 h after cerebral ischemia. In addition, the levels of tryptophan in the MCAO groups were all obviously increased at 3 h, 6 h, 12 h, and 24 h after ischemia compared to the sham groups (*p* < 0.01). This latter finding was consistent with our previous study results which demonstrated an obvious increase in the tryptophan level in rat plasma 12 h after MCAO-induced cerebral ischemia. The line charts of the concentration-ischemia time for the ten AAs in CSF of all the groups are shown in Figure 5.

After pretreatment of BNC, these perturbations of AAs in the imbalanced network of CI could be partly reversed at different time points after MCAO. Until 24 h after MCAO, the nine AA biomarkers of MCAO_3-24h_ proved to be close to normal after administration of BNC (BNC vs. MCAO, *p* < 0.05). Particularly, in the BNC_24h_-treated group, seven of the eight AA biomarkers of MCAO_24h_, i.e., alanine, valine, leucine, glutamine, phenylalanine, tyrosine, and tryptophan, were close to normal compared to the MCAO_24h_ group (glutamine *p* < 0.05, all others *p* < 0.01). As for glutamate, although the level of glutamate in the BNC-treated_24h_ group did not obviously change compared to the MCAO_24h_ group, the level of glutamate in the BNC-treated_6h_ and BNC-treated_12h_ groups proved to be normal compared to that in the corresponding MCAO groups (*p* < 0.01). These findings suggest that the therapeutic effects of BNC on cerebral ischemia were partly due to interferences with the AA metabolism of the imbalanced network.

### 3.5. Changes of Proteins Associated with CI

Based on the CI imbalanced network of AAs-enzymes-proteins, we found a total of 36 enzymes regulating the generation of the nine BNC-mediated AA biomarkers. According to the value of “degree” (degree > 2), which is defined as the number of links to AAs, four regulatory enzymes of GOT1 (regulating Glu, Phe, and Tyr), GOT2 (regulating Glu, Phe, and Tyr), TAT, and AADC (regulating Try, Phe, and Tyr) were selected. Furthermore, in the AAs regulated by the four enzymes, glutamate (Glu) as a major excitatory amino acid features an important role in the excitatory death of neurons after MCAO. Tryptophan (Try) was the only AA exhibiting significant changes out of the ten detected AAs at all time points after MCAO.

**Figure 6:**
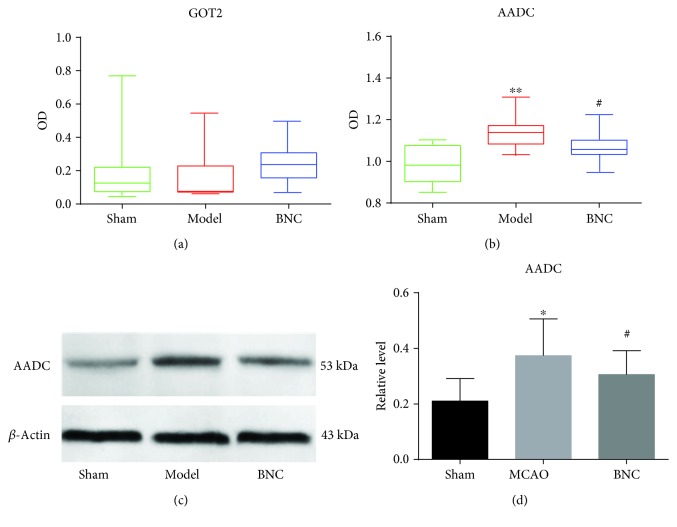
Verification of the enzymes regulated by BNC against CI in CSF based on ELISA and western blotting. ELISA test for GOT2 (a) and AADC (b); western blotting test for AADC (c); three individual samples were analyzed in each group. Results were analyzed by one-way ANOVA. ^∗^ *p* < 0.05 and ^∗∗^ *p* < 0.01 in the MCAO group versus the sham group; ^#^ *p* < 0.05 in the BNC group versus the MCAO group.

It is well-known that an imbalanced biological disease network should be dynamic. In the biological information flux, protein changes should take place earlier than metabolite changes. In this study, although major AA changes were observed 24 h after MCAO, related enzyme or protein changes may take place far sooner. Therefore, the CSF samples of 3, 6, 12, and 24 h after MCAO were combined and used for ELISA assays. As for TAT and GOT1, the test results of CSF samples were both negative, possibly due to concentrations that were too low for detection in CSF. The contents of AADC and GOT2 in CSF for the different groups are shown in Figure 6. For GOT2, no significant differences between the sham group, MCAO group, and BNC-treated group could be observed. The level of AADC in the MCAO group exhibited a significant increase compared to the sham group (*p* < 0.01). Moreover, compared to the MCAO group, the content of AADC in the BNC-treated model was found to be significantly decreased (*p* < 0.05) and moved towards a normal state. In order to verify this result, the expression of AADC in CSF for the sham, MCAO, and BNC-treated groups at 12 h after MCAO was further quantified by western blotting. The changes of the AADC level between the sham, MCAO, and BNC-treated groups measured by western blotting were the same as those measured by ELISA (cf. Figures 6(b) and 6(c)). These results implied that AADC may play a critical role in the protection against cerebral ischemic for BNC.

Previous studies have shown that AADC plays a significant role in brain development and is functionally associated with several neurologic disorders such as Parkinson's and Alzheimer's disease [47–49]. Similar to our results, Bauer et al. found that AADC activity was increased after hypoxia/hypercapnia in newborn piglets [50]. However, this report was the first to describe the relationship between MCAO-induced cerebral ischemia and AADC activity. It is known that the general function of AADC is involved in carboxylase activity. In particular, except for L-5-hydroxytryptophan to serotonin and L-tryptophan to tryptamine, AADC may catalyze the decarboxylation of L-3,4-dihydroxyphenylalanine (DOPA) to dopamine (DA). It is well-known that after cerebral ischemia, a massive release of DA from ischemic neurons can be observed, exhibiting neurotoxic effects and directly contributing to cell death [51–54]. Thus, AADC may serve as a promising target for the treatment of ischemic stroke and may help in the discovery of new drug candidates.

In addition, in the CI imbalanced network (cf. Figure 1), four proteins involving the synaptic vesicular amine transporter (Slc18a2), glycogen phosphorylase (pygb), alcohol dehydrogenase type 3 gene (Adh1c), and aldehyde dehydrogenase 2 (Aldh2) exhibited protein-protein interactions with AADC. Although some reports can be found in the literature on the correlation between these proteins and cerebral ischemia, comprehensive data on the relationship between AADC and the corresponding interactional proteins requires further studies.

## 4. Conclusions

In this study, an imbalanced network, containing metabolites of AAs, key regulatory enzymes, and proteins associated with CI, was constructed offering the possibility to further understand the pharmacological mechanisms of BNC acting on CI. Furthermore, a novel analytical method for the detection of ten AAs without derivatization in CSF was developed by UPLC-QQQ-MS to further validate the imbalanced networks and the therapeutic effects of BNC from the levels of metabolites. The method proved to be rapid, sensitive, accurate, and reproducible within acceptable limits and could be used to analyze AAs in CSF.

Based on a middle cerebral artery occlusion (MCAO) rat model, the efficacy of BNC was confirmed by reducing cerebral infarction and improving the neurological behavior scores. Then, the dynamic levels of the amino acids in CSF 3, 6, 12, and 24 hours after MCAO were analyzed. An overview based on score plots of principal component analysis showed that with the extension of ischemic time, the distinction observed between the groups (sham, MCAO, and BNC-treated group) became more obvious. There were 2, 2, 5, and 8 AAs exhibiting significant content changes in the MCAO_3h_, MCAO_6h_, MCAO_12h_, and MCAO_24h_ groups compared with the corresponding sham groups, respectively. Until 24 h after MCAO, except for taurine, there were accumulated nine AA biomarkers exhibiting significant changes in the MCAO group. After BNC treatment, all nine AA biomarkers demonstrated a tendency for returning to baseline values.

Based on the imbalanced network of CI, four related enzymes of AADC, GOT1, GOT2, and TAT, regulating the generation of BNC-mediated AA biomarkers, were selected as well as tested using ELISA and western blotting. Finally, the level of AADC in the MCAO group exhibited a significant increase compared to the sham group (*p* < 0.05). Moreover, compared to the MCAO group, the AADC content in the BNC-treated model was shown to be significantly decreased (*p* < 0.05) and was demonstrated to move towards a normal state. This result indicated that AADC proved to be one of the putative targets for BNC in the protection against cerebral ischemia.

In summary, the developed analytical methods for the detection of amino acids provided potential tools for further related disease studies. Moreover, the results obtained throughout this study may help develop novel strategies to explore the mechanism of cerebral ischemia and may also be useful to discover potential targets for BNC and other related drug candidates.

## Figures and Tables

**Table 1:** The MS parameters for detecting 10 amino acids in CSF in ESI^+^ mode.

No.	Amino acids	Formula	Parent (m/z)	Daughter (m/z)	Cone (V)	Collision (V)	Retention time (min)
1	Tryptophan	C_11_H_12_N_2_O_2_	204.96	187.82	12	8	2.43
2	Tyrosine	C_9_H_11_NO_3_	181.90	135.84	20	12	3.15
3	Phenylalanine	C_9_H_11_NO_2_	165.96	119.83	18	10	2.46
4	Glutamate	C_5_H_9_NO_4_	147.90	83.84	20	12	4.32
5	Glutamine	C_5_H_10_N_2_O_3_	146.90	83.86	26	14	5.03
6	Isoleucine	C_6_H_13_NO_2_	131.90	85.89	28	8	2.51
7	Leucine	C_6_H_13_NO_2_	131.90	85.89	20	8	2.41
8	Taurine	C_2_H_7_NO_3_S	125.84	107.82	40	10	3.32
9	Valine	C_5_H_11_NO_2_	117.90	71.88	24	8	2.91
10	Alanine	C_3_H_7_NO_2_	89.90	43.89	18	6	3.85
11	Phenylalanine-d5 (IS)	C_9_H_6_D_5_NO_2_	171.16	125.03	22	12	2.46

**Table 2:** Linear range and correlation coefficient and sensitivity of 10 amino acids in ESI^+^ mode.

Amino acids	Linear range (ng/mL)	r	LOD			LOQ		
			Content	S/N average	RSD% (*n* = 5)	Content	S/N average	RSD% (*n* = 5)
Tryptophan	6.04-386.25	0.9985	100 pg/mL	5.27	9.67	1000 pg/mL	14.58	10.89
Tyrosine	2.50-160.00	0.9844	15 pg/mL	4.10	14.37	150 pg/mL	12.61	11.71
Phenylalanine	0.98-62.40	0.9995	2 pg/mL	12.02	12.53	20 pg/mL	13.18	16.91
Glutamate	37.19-2380.00	0.9995	50 pg/mL	6.44	13.31	500 pg/mL	14.83	12.77
Glutamine	177.19-11340.00	0.9834	5 pg/mL	4.64	19.54	50 pg/mL	17.72	10.14
Isoleucine	1.23-78.60	0.9960	3.5 pg/mL	5.04	15.19	35 pg/mL	12.97	5.13
Leucine	0.90-57.50	0.9995	0.7 pg/mL	6.57	16.79	7 pg/mL	14.30	12.86
Taurine	43.24-2767.50	0.9980	110 pg/mL	3.16	13.19	1100 pg/mL	17.05	14.49
Valine	1.58-101.00	0.9995	3.5 pg/mL	4.65	12.14	35 pg/mL	10.95	9.52
Alanine	18.28-1170.00	0.9995	100 pg/mL	5.35	14.55	1000 pg/mL	12.69	15.76

S/N was calculated as RMS (root mean square) S/N.

**Table 3:** Precisions, repeatability, accuracy, and stability data for the ten AAs in CSF.

Amino acids	Precision		Repeatability RSD% (*n* = 6)	Accuracy		Stability	
	Intraday RSD% (*n* = 6)	Interday RSD% (*n* = 7)		Average recovery (*n* = 6)	Recovery RSD%	Three freeze-thaw stability RSD% (*n* = 6)	Short-term stability RSD% (*n* = 6)
Tryptophan	2.07	1.98	4.16	81.27	2.70	2.19	4.16
Tyrosine	1.95	2.70	0.59	80.30	5.34	2.38	0.59
Phenylalanine	1.87	2.04	4.42	80.47	3.46	1.12	4.42
Glutamate	2.85	1.78	3.14	104.51	2.07	1.15	3.14
Glutamine	2.30	2.44	3.93	115.29	2.24	1.37	3.93
Isoleucine	2.80	2.39	7.99	113.33	2.63	1.81	1.79
Leucine	2.99	2.99	5.75	88.64	5.55	1.18	4.75
Taurine	0.87	2.45	3.96	87.25	2.18	1.59	3.96
Valine	2.47	1.75	7.86	94.77	3.17	2.19	5.86
Alanine	2.91	2.09	2.00	90.78	7.19	1.75	2.00

**Table 4:** The content (pg/*μ*L) of 10 amino acids in CSF in different groups (*n* = 6).

Amino acids	Groups	3 h	6 h	12 h	24 h
Alanine	Sham	11.26 ± 0.78	16.04 ± 3.35	20.07 ± 3.05	13.29 ± 1.98
	Model	10.73 ± 1.36	16.25 ± 2.30	19.71 ± 3.50	19.26±3.36^∗∗^
	BNC	11.58 ± 1.35	15.64 ± 3.27	25.11 ± 4.65	12.08 ± 1.90^##^
Valine	Sham	2.44 ± 0.21	2.80 ± 0.22	3.68 ± 0.69	2.69 ± 0.26
	Model	2.54 ± 0.57	2.86 ± 0.49	2.91 ± 0.63^∗^	3.43±0.43^∗∗^
	BNC	2.84 ± 0.29	2.72 ± 0.38	5.18 ± 1.00	2.25 ± 0.42^##^
Taurine	Sham	5.28 ± 1.37	4.51 ± 1.04	7.45 ± 1.06	5.61 ± 0.89
	Model	4.59 ± 0.46	4.90 ± 1.05	7.15 ± 1.06	5.82 ± 0.72
	BNC	5.24 ± 0.54	4.47 ± 0.77	8.95 ± 1.83	4.63 ± 0.91
Leucine	Sham	5.52 ± 0.26	5.75 ± 0.96	11.06 ± 2.14	6.16 ± 0.68
	Model	5.34 ± 0.52	5.96 ± 0.75	11.71 ± 2.19	7.67±0.99^∗∗^
	BNC	6.23 ± 0.56	5.73 ± 1.01	15.46 ± 2.27	4.87 ± 0.83^##^
Isoleucine	Sham	0.88 ± 0.12	1.60 ± 0.12	1.69 ± 0.39	1.45 ± 0.17
	Model	0.81 ± 0.09	1.60 ± 0.32	1.23 ± 0.20^∗^	1.42 ± 0.10
	BNC	1.11 ± 0.15	1.53 ± 0.27	2.23 ± 0.29^#^	1.02 ± 0.14
Glutamine	Sham	555.08 ± 41.94	714.20 ± 38.70	970.05 ± 47.48	618.39 ± 95.07
	Model	575.64 ± 55.92	786.44 ± 133.18	1026.90 ± 72.82^∗^	791.02±127.71^∗∗^
	BNC	584.33 ± 40.38	760.09 ± 59.41	1081.74 ± 79.86	625.94 ± 118.44^#^
Glutamate	Sham	1.90 ± 0.23	1.50 ± 0.11	1.64 ± 1.09	2.04 ± 0.33
	Model	1.99 ± 0.43	3.80 ± 0.33^∗^	5.07±1.08^∗∗^	2.89 ± 0.21^∗^
	BNC	1.60 ± 0.12	2.49 ± 0.22^#^	1.65 ± 0.33^##^	3.31 ± 0.27
Phenylalanine	Sham	7.80 ± 0.62	9.62 ± 1.73	16.27 ± 2.74	8.98 ± 1.33
	Model	8.73 ± 1.25	9.89 ± 1.03	15.75 ± 3.09	12.54±2.06^∗∗^
	BNC	9.21 ± 0.76	10.71 ± 1.84	18.96 ± 2.22	7.15 ± 1.13^##^
Tyrosine	Sham	8.06 ± 0.89	11.30 ± 1.72	12.32 ± 2.23	9.66 ± 1.60
	Model	10.86±1.79^∗∗^	10.83 ± 2.50	13.58 ± 3.09	13.24±1.07^∗∗^
	BNC	11.47 ± 1.22	11.96 ± 2.24	14.89 ± 2.34	9.57 ± 1.26^##^
Tryptophan	Sham	3.48 ± 0.32	3.73 ± 0.75	7.07 ± 1.29	9.66 ± 0.57
	Model	5.03±0.84^∗∗^	4.81±1.02^∗∗^	9.43±1.86^∗∗^	13.24±0.76^∗∗^
	BNC	5.49 ± 0.41^##^	5.09 ± 0.43	11.86 ± 1.74	9.57 ± 0.42^##^

^∗^ *p* < 0.05, ^∗∗^ *p* < 0.01, and ^∗∗∗^ *p* < 0.001 in the MCAO group versus the sham group; ^#^ *p* < 0.05, ^##^ *p* < 0.01, and ^###^ *p* < 0.001 in the BNC group versus the MCAO group.

## Data Availability

The data used to support the findings of this study were included within the article and the supplementary information file.

## Supplementary Materials

Supplementary Materials Table S1: the summary of amino acids, regulatory enzymes, and proteins related to cerebral ischemia.
