# Supplementary material for: A Metabonomics Investigation into the Therapeutic Effects of BuChang NaoXinTong Capsules on Reversing the Amino Acid-Protein Interaction Network of Cerebral Ischemia
Source: Oxid Med Cell Longev. 2019 Mar 20;2019:7258624. doi: 10.1155/2019/7258624 (PMC6446104; doi:10.1155/2019/7258624)
Supplement: Supplementary Materials — Table S1: the summary of amino acids, regulatory enzymes, and proteins related to cerebral ischemia. [file 7258624.f1.docx]

**Supplementary materials**

In order to investigate the relationship between metabolites and proteins after cerebral ischemia, a cerebral ischemia imbalanced network of AAs-enzymes-proteins was constructed. According to the protocols including the four main steps which had been written detailedly in the manuscript 2.2, the AAs, enzymes and proteins which are related to cerebral ischemia was filtrated and summarized in the following Table S1. Based on the data in the Table S1, the visual networks of AAs-enzymes-proteins (Figure 1 in the manuscript) was constructed by Cytoscape (Version 13.6)

Table S1 The summary of amino acids, regulatory enzymes and proteins related to cerebral ischemia

| AA | Regulatory enzyme | Protein |
| --- | --- | --- |
| Alanine | Abat | Gad2  Gatm |
|  | Agxt2 | Shmt1  Gatm  Nos1  Cad |
|  | Agxt | Shmt2  Gldc |
|  | Gpt | Gldc |
| Dopamine | Maob | Aldh2  Adh1c |
| Serine | Cbs | Nos1  Nos2 |
|  | Agxt | Shmt2  Gldc |
|  | Shmt1 | Pfas  Gatm  Gldc  Mthfr  Agxt2  Cad  Shmt2 |
|  | Sdsl | Gfpt2 |
|  | Shmt2 | Gldc  Mthfr  Agxt2  Cad  Pfas  Gatm  Shmt1 |
| Tyronsine | Th | Nrg1  Abcc8  Mpl |
|  | Tat | Mif  Gldc |
|  | Aadc | RGD1308234  Slc18a2  Slc18a2  Pygb  Adh1c  Aldh2 |
|  | Got1 | Mif  Gad2 |
|  | Got2 | Mif  Gad2  Lpl |
| Citrulline | Ass1 | Nos3  Nos2  Nos1  Gad2 |
|  | Nos2 | Arg1  Ass1  Otc  Gatm  Il12b  Nos1  Hmox1  Tnf  Nos3  Akt1  Prkce  Stat4  Il10  Cbs  Tlr4  Il4  Jak2 |
|  | Otc | Nos3  Nos2  Nos1 |
|  | Nos1 | Arg2  Ass1  Arg1  Camk2d  Ryr2  Otc  Gatm  Grin2b  Grin2c  Grin1  Ryr3  Akt1  Dlg4  Gucy1a3  Nos3  Il12b  Prkce  Agt  Cbs  Tnf  Hmox1 |
|  | Nos3 | Ass1  Otc  Gatm  Ace  Crp  Epo  Akt1  Arg1  Arg2  Camk2d  Nos2  Agt  Nos1  Vegfa  Prkce  Fgf2  COX2 |
| GABA | Abat | Gad2  Gatm |
|  | Gatm | Gad1  Arg1  Gamt  Arg2  Nos2  Shmt2  Shmt1  Agxt2  RGD1308234  Nos3  Gldc  Nos1  Aldh2  Glul  Glud1  Abat  Gad2  Gls2 |
| Threonine | Sdsl | Gfpt2 |
| Glutamate | Gls2 | Aldh2  Gatm  Gldc  RGD1308234 |
|  | Gls | Gad2  Aldh2  Gldc  RGD1308234 |
| Trypyophan | Aadc | RGD1308234  Slc18a2  Slc18a2  Pygb  Adh1c  Aldh2 |
| Serotonin | Maob | Aldh2  Adh1c |
|  | Aadc | RGD1308234  Slc18a2  Slc18a2  Pygb  Adh1c  Aldh2 |
| Glycine | Agxt2 | Shmt1  Gatm  Nos1  Cad |
|  | Gatm | Gad1  Arg1  Gamt  Arg2  Nos2  Shmt2  Shmt1  Agxt2  RGD1308234  Nos3  Gldc  Nos1  Aldh2  Glul  Glud1  Abat  Gad2  Gls2 |
|  | Agxt | Shmt2  Gldc |
|  | Gss | Gpx1  Gstm3 |
|  | Shmt2 | Gldc  Mthfr  Agxt2  Cad  Pfas  Gatm  Shmt1 |
|  | Shmt1 | Pfas  Gatm  Gldc  Mthfr  Agxt2  Cad  Shmt2 |
|  | Grin3b | Dlg4 |
|  | Grin2a | Dlg4 |
|  | Grin2c | Dlg4  Nos1 |
|  | Glyat | Keg1 |
|  | Shmt2 | Gldc  Mthfr  Agxt2  Cad  Pfas  Gatm  Shmt1 |
| Phenylalanine | Th | Nrg1  Abcc8  Mpl |
|  | Tat | Mif  Gldc |
|  | Ddc | RGD1308234  Slc18a2  Slc18a2  Pygb  Adh1c  Aldh2 |
|  | Got1 | Mif  Gad2 |
|  | Got2 | Mif  Gad2  Lpl |
| Glutamine | Asns | Cad  Gad2 |
|  | Glul | Gad2  Gldc  Aldh2  RGD1308234  Gatm  Slc1a2 |
|  | Gls2 | Aldh2  Gatm  Gldc  RGD1308234 |
|  | Gls | Gad2  Aldh2  Gldc  RGD1308234 |
|  | Pfas | Shmt2  Shmt1  Ctps  Gldc |
|  | Abat | Gad2  Gatm |
|  | Tat | Mif  Gldc |
|  | Got1 | Mif  Gad2 |
|  | Got2 | Mif  Gad2  Lpl |
|  | Agxt2 | Shmt1  Gatm  Nos1  Cad |
|  | Oat | Cad |
|  | Asns | Cad  Gad2 |
|  | Glul | Gad2  Gldc  Aldh2  RGD1308234  Gatm  Slc1a2 |
|  | Gls2 | Aldh2  Gatm  Gldc  RGD1308234 |
|  | Gls | Gad2  Aldh2  Gldc  RGD1308234 |
|  | Glud1 | Aldh2  Gatm  Gad2  Gldc  RGD1308234 |
|  | Gad2 | Abat  Gad1  Glud1  Aldh2  Asns  Got2  Got1  Ass1  Gatm  Glul  Cad  Gls  Stx1a  RGD1308234  Il4i1  Gldc |
|  | Gad1 | Cad  Gad2  Aldh2  Stx1a  RGD1308234  Gldc  Gatm  Slc17a7 |
|  | Agxt | Shmt2  Gldc |
|  | Gss | Gpx1  Gstm3 |
|  | Gpt | Gldc |
|  | Pfas | Shmt2  Shmt1  Ctps  Gldc |
|  | Aldh18a1 | Aldh2 |
|  | Grin3b | Dlg4 |
|  | Grin2a | Dlg4 |
|  | Grin2c | Dlg4  Nos1 |
| Histidine | Aadc | RGD1308234  Slc18a2  Slc18a2  Pygb  Adh1c  Aldh2 |
| Aspartate | Got1 | Mif  Gad2 |
|  | Got2 | Mif  Gad2  Lpl |
|  | Acy1 | Cad |
|  | Asns | Cad  Gad2 |
|  | Ass1 | Nos3  Nos2  Nos1  Gad2 |
|  | Glud1 | Aldh2  Gatm  Gad2  Gldc  RGD1308234 |
|  | Gad2 | Abat  Gad1  Glud1  Aldh2  Asns  Got2  Got1  Ass1  Gatm  Glul  Cad  Gls  Stx1a  RGD1308234  Il4i1  Gldc |
|  | Gad1 | Cad  Gad2  Aldh2  Stx1a  RGD1308234  Gldc  Gatm  Slc17a7 |
|  | Ass1 | Nos3  Nos2  Nos1  Gad2 |
| Nicotinuric acid | Glyat | Keg1 |
| Homocysteine | Cbs | Nos1  Nos2 |
| Ornithine | Arg1 | Nos3  Gatm  Il10  Il4  Nos2  Nos1  Cad |
|  | Oat | Cad |
|  | Gatm | Gad1  Arg1  Gamt  Arg2  Nos2  Shmt2  Shmt1  Agxt2  RGD1308234  Nos3  Gldc  Nos1  Aldh2  Glul  Glud1  Abat  Gad2  Gls2 |
|  | Acy1 | Cad |
|  | Aldh18a1 | Aldh2 |
|  | Otc | Nos3  Nos2  Nos1 |
|  | Arg2 | Nos3  Gatm |
| Arginine | Arg1 | Nos3  Gatm  Il10  Il4  Nos2  Nos1  Cad |
|  | Gatm | Gad1  Arg1  Gamt  Arg2  Nos2  Shmt2  Shmt1  Agxt2  RGD1308234  Nos3  Gldc  Nos1  Aldh2  Glul  Glud1  Abat  Gad2  Gls2 |
|  | Ass1 | Nos3  Nos2  Nos1  Gad2 |
|  | Nos2 | Arg1  Ass1  Otc  Gatm  Il12b  Nos1  Hmox1  Tnf  Nos3  Akt1  Prkce  Stat4  Il10  Cbs  Tlr4  Il4  Jak2 |
|  | Nos1 | Arg2  Ass1  Arg1  Camk2d  Ryr2  Otc  Gatm  Grin2b  Grin2c  Grin1  Ryr3  Akt1  Dlg4  Gucy1a3  Nos3  Il12b |
|  | Nos3 | Ass1  Otc  Gatm  Ace  Crp  Epo  Akt1  Arg1  Arg2  Camk2d  Nos2  Agt  Nos1  Vegfa  Prkce  Fgf2  COX2 |
|  | Arg2 | Nos3  Gatm |
| Taurine | Cad2 | Shmt1  Shmt2  Cad |
| Valine | Comt | Adh1c  Slc18a2  Esr1  Ankk1  Ace  Mthfr |
|  | Bcat1 | Ccbl1  Gldc  Gad2  Dlg4 |
|  | Bcat2 | Gldc  Gad2  Dlg4 |
| leucine | Bcat1 | Ccbl1  Gldc  Gad2  Dlg4 |
|  | Bcat2 | Gldc  Gad2  Dlg4 |
| Isoleucine | Bcat1 | Ccbl1  Gldc  Gad2  Dlg4 |
|  | Bcat2 | Gldc  Gad2  Dlg4 |
